# Tip Surgery in Dorsal Preservation Rhinoplasty: The Effect of Modified Low Septal Strip Septoplasty on Tip Plasty

**DOI:** 10.1007/s00266-024-04218-4

**Published:** 2024-07-08

**Authors:** Vasfi Çelik, Yavuz Tuluy, Gökçen Çakır Bozkurt

**Affiliations:** 1 Private Practice, Plastic, Reconstructive and Aesthetic Surgery, Mersin, Turkey; 2 Department of Plastic, Reconstructive and Aesthetic Surgery, İzmir Bakırçay University Çiğli Training and Research Hospital, İzmir, Turkey; 3 Department of Plastic, Reconstructive and Aesthetic Surgery, Manisa City Hospital, Manisa, Turkey

**Keywords:** Dorsal preservation rhinoplasty, Low septal strip septoplasty, Tip surgery, Septum fixation.

## Abstract

**Background:**

The aim in tip surgery is to provide rotation, derotation, projection and deprojection. In this study, we aimed to show the effects of modified low septal strip septoplasty, septal extension graft, TIG technique and additional maneuvers on tip shape in dorsal preservation rhinoplasty (DPR) and to discuss our clinical results.

**Patients and Methods:**

One hundred eighty-nine patients who underwent DPR with modified low septal strip septoplasty between November 2021 and August 2023 were included in the study. Demographic data, complications, revision surgeries and follow-up periods of the patients were analyzed retrospectively.

**Results:**

The mean age of the patients is 29.58±9.04 (17-65). The mean follow-up period was 14, 50±2,98 months. Complications were observed in 1.1% of the patients (*n*=2/189). Revision surgery was performed in all these patients. Residual hump in 2 were observed and dorsum rasping was performed under local anesthesia. No tip revision was performed on any patient.

**Conclusions:**

A strong tip fixation is achieved with the modified low septal septoplasty technique described in this publication, and when combined with septal extension graft, tongue in groove technique and other suture techniques, an effective and permanent tip plasty can be performed in DPR.

**Level of Evidence II:**

This journal requires that authors assign a level of evidence to each article. For a full description of these Evidence-Based Medicine ratings, please refer to the Table of Contents or the online Instructions to Authors www.springer.com/00266.

**Supplementary Information:**

The online version contains supplementary material available at 10.1007/s00266-024-04218-4.

## Introduction

Dorsal preservation rhinoplasty is one of the techniques popularized by nose surgeons in recent years. The nose is shaped by preserving the dorsum without the need for dorsum reconstruction. Tip surgery can be performed at the beginning or at the end of the surgery and varies according to the surgeon's preference. There are many techniques for tip surgery, and it is performed by using sutures or cartilage grafts. Although interdomal, transdomal sutures, cap graft, onlay tip graft, shield graft and strut graft are techniques known to many surgeons, the approach to tip surgery varies widely. The aim in tip surgery is to provide rotation, derotation, projection and deprojection. One of the grafts used in tip surgery is septal extension grafts. Septal extension grafts are generally used to lengthen the nose and are also used to provide tip projection and rotation. It has been used frequently in nose surgery since they were defined by Byrd [1]. It is divided into 3 types. These are paired spreader type, paired batten type and direct extension type. In modern rhinoplasty, septal extension grafts have been widely used in tip reshaping as well as in patients with short noses. There are modified uses of septal extension grafts in the literature [2–6]. Suturing techniques are commonly used in tip surgery in combination with cartilage grafts. One of the suturing techniques used in tip surgery is the tongue-in-groove (TIG) technique. It was first described by Rethi and then popularized by Kridal in 1999 [7]. Tip projection and rotation is achieved by performing a columellar set back with the TIG technique. The original TIG technique has some disadvantages. These are shortening of the nose and stiffness in nasal tip movements [8]. Therefore some modifications were described to overcome the disadvantages [8–10]. Success in nasal tip surgery is only possible with a strong septum. A septum that is far from the midline or curved to the right or left will negatively affect the tip plasty. In dorsal preservation rhinoplasty (DPR), septoplasty is performed using low-middle or high septal strip techniques. In the low septal strip septoplasty technique, cartilage and bone resection is performed from the dorsum, vertical line, and the base of the septum, and let down or push down operation is performed. Strong fixation of the septum during septoplasty will positively affect the tip surgery and ensure longer and more permanent results. Inadequate fixation may lead to a droopy tip, loss of tip projection and rotation, or even right and left deviations in the columella. Therefore, we focus on the septum fixation in combination with cartilage grafts and suturing techniques to achieve better tip surgery in DPR. In this study, we aimed to show the effects of modified low septal strip septoplasty, septal extension graft, TIG technique and additional maneuvers on tip shape in DPR and to discuss our clinical results.

## Patients and Methods

One hundred eighty-nine patients who underwent preservation rhinoplasty with modified low septal strip septoplasty between November 2021 and August 2023 were included in the study. This research was conducted per the Declaration of Helsinki guidelines. All the patients have provided written informed consent for the surgery and use of their data. Closed dorsal preservation rhinoplasty was performed with modified low septal strip septoplasty. All cases were primary rhinoplasty. Demographic data, complications, revision surgeries and follow-up periods of the patients were analyzed retrospectively (Table 1).

**Table 1:** Demographic characteristics of the patients and the results

		Modified low septal strip septoplasty (n=189)
Gender	Female	147 (77,8)
	Male	42 (22,2)
Age	*Mean±Sd*	29,58±9,04
	*Median (Min-Max)*	27 (17-65)
Complication	None	187 (98,9)
	Yes	2 (1,1)
Recurrent hump	None	189 (100)
	Yes	0 (0)
Revision Surgery	None	187 (98,9)
	Yes	2 (1,1)
Follow-up(month)	*Mean±Sd*	14,50±2,98
	*Median (Min-Max)*	14 (9-19)

### Surgical Technique

The operation was performed under general anesthesia and the systolic blood pressure was reduced to 80-90 mm Hg. Local anesthetic infiltration was done with adrenaline and Marcaine. The septum and caudal dorsum were exposed with the bilateral hemitransfixation incision, while the lateral, middle, and medial cruses of LLC, nasal dorsum and lateral walls of the nose were exposed with the infracartilaginous incision. Septum dissection was revealed in the supraperichondreal plane, preserving the perichondrium over the septum. Following modified low septal strip septoplasty, the junction of the ULC and nasal bone was released by preserving the keystone. In modified low septal septoplasty, 2 mm of cartilage was left on the vomer and maxilla for suturing of the quadrangular cartilage. Lateral, medial, and median osteotomies were performed with the help of a piezotome. The nose was freed by moving the bone pyramid to the right and left. In patients who were planned to let down operation, a 2 mm bone strip was removed at the bases of the bone pyramid with a piezotome. The hump was evaluated by pulling the cartilage septum caudal first inferiorly and then anteriorly with forceps. Triangular cartilage that overlaps on the vomer from the quadrangular cartilage was removed and taken into isotonic solution to be used as a septal extension graft. The quadrangular cartilage was fixed to the 2 mm cartilage left on the vomer with 3/0 pds in the cranial to caudal direction (video 1). After fixation with 3 sutures, dorsum was checked again. In patients with prominent kyphotic dorsum, fine rasping was performed on the dorsum with a piezotome. After fixation, the excess triangular cartilage remaining on the caudal septum was removed, and the septum was sutured to the cartilage left on the anterior nasal spine with 3/0 pds, and the fixation was completed. Triangular cartilage was taken into isotonic solution to be used as a septal extension graft. We aimed to prevent residual and recurrent hump by performing septum surgery in this way. The strong septum also serves as an important support for tip surgery. We think that we prevent the loss of nasal tip projection and derotation that can be seen in the late period with this technique. Lateral crus surgery was completed by excising the excess cephalic part of the LLC or suturing the excess cartilage to lateral crus by designing a slide under flap in patients who was needed to strengthen the lateral crus (video 2). Slide under flap was also used to correct the deformity in patients with convexity or concavity in the lateral crus. Hemitransdomal sutures were placed bilaterally for tip definition. Triangular cartilage grafts taken from the septum were fixed to the right and left of the caudal septum. Ligaments located caudal to the medial crus in the columella were released anteriorly and posteriorly with the help of scissors. Thus, it was aimed to prevent the anterior retraction of the philtral column and short columella, especially seen in tension nose patients. Later, both domes were fixed to septal extension grafts with 5/0 pds. Tip surgery was completed by placing a membranous tongue-in groove suture. In patients with long medial crus, the over projection was corrected by overlapping the medial crus. After the repair of the incisions, the operation was terminated by placing a silicone nose pad, bandage, and splint.

## Results

**Fig. 1:**
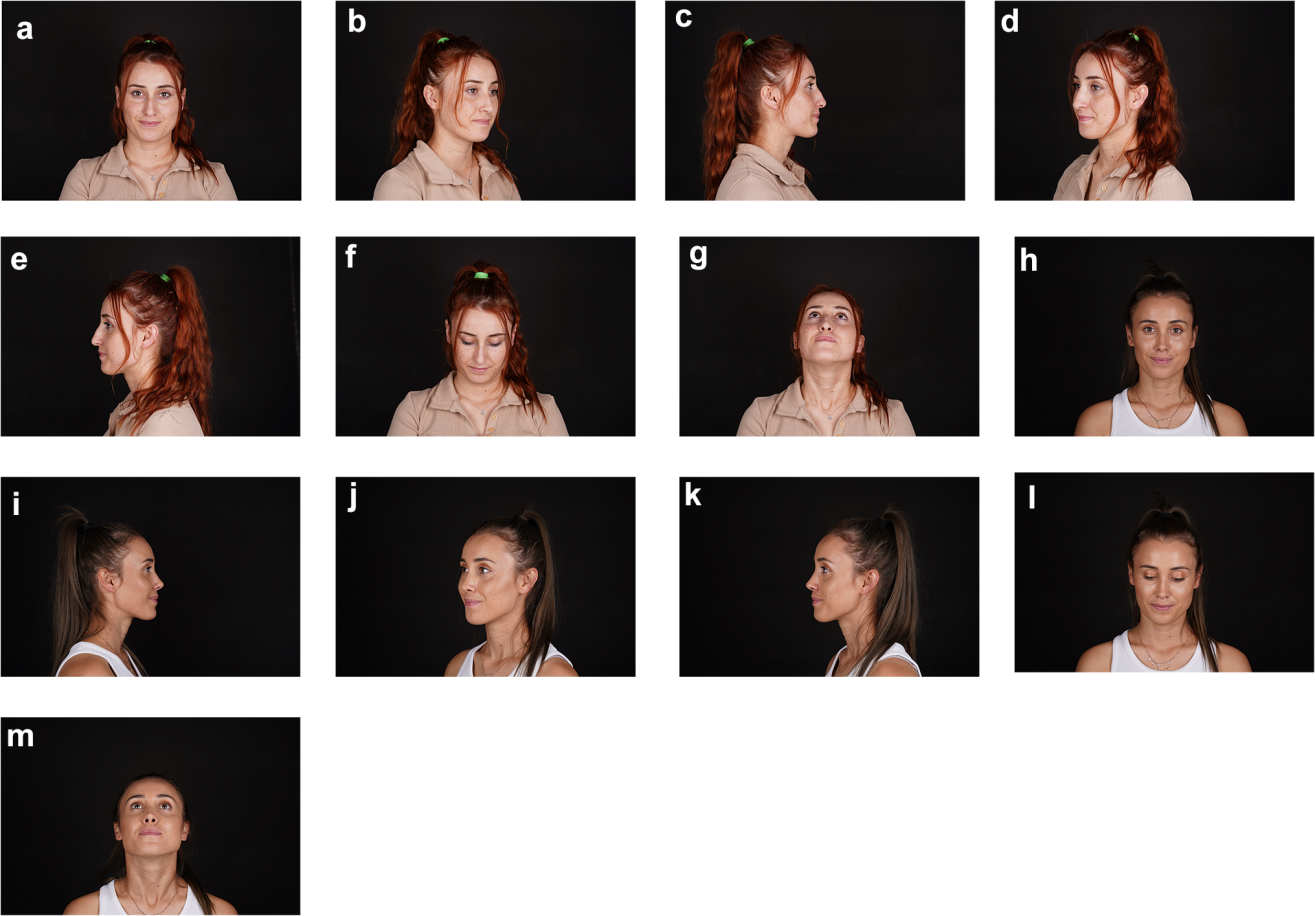
**a**–**m** Postoperative first year result of a 32-year-old patient

**Fig. 2:**
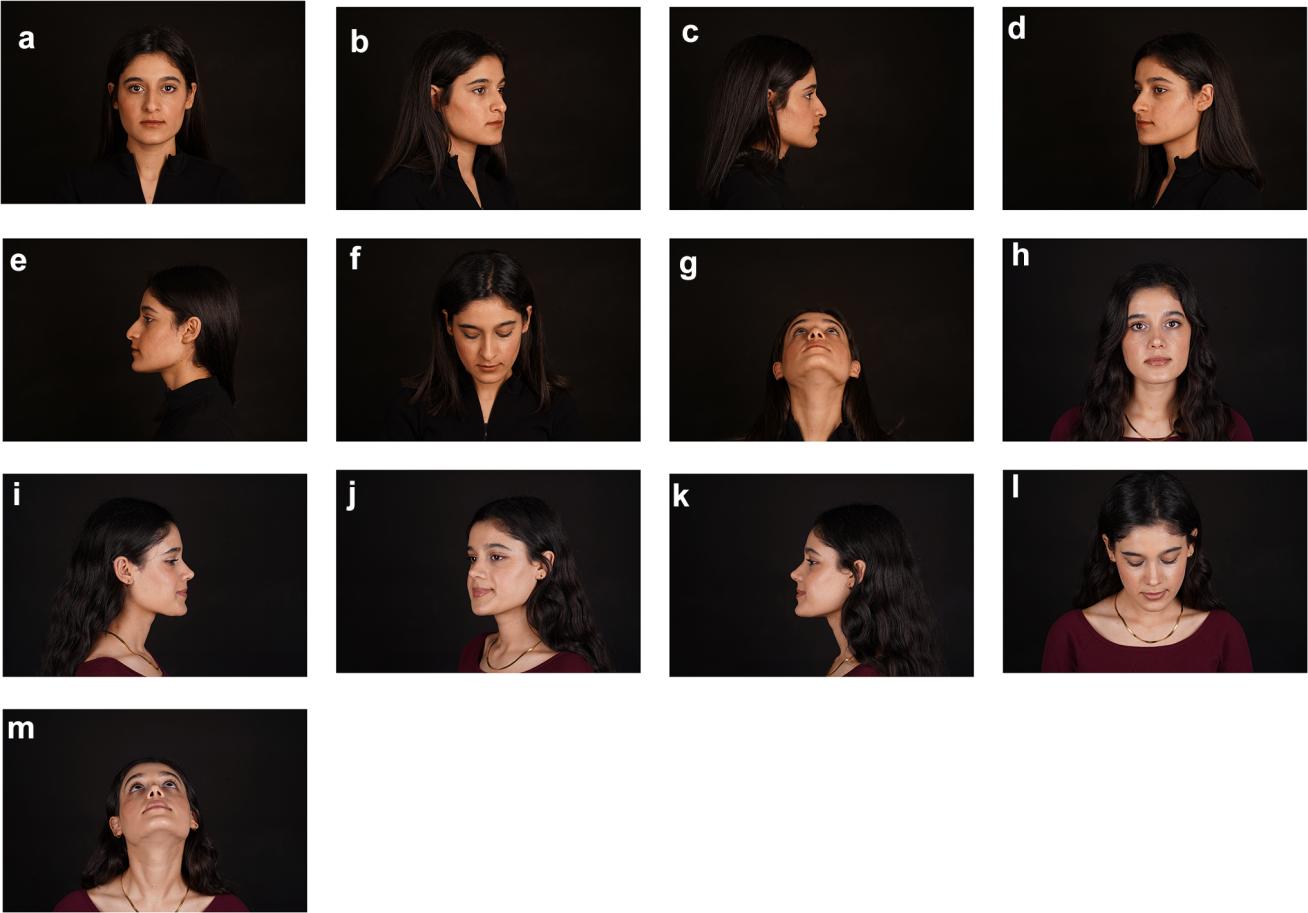
**a**–**m** Postoperative first year result of a 22-year-old patient

The surgeries were performed under general anesthesia. 77.8% (*n*=147) of the patients were female and 22.2% (*n*=42) were male (Table 1). The mean age of the patients is 29.58±9.04 (17-65). The mean follow-up period was 14, 50±2, 98 months. Patients were discharged on the first postoperative day. Nasal splints and silicone nose pads were removed on the 5^th^ postoperative day. Complications were observed in 1.1% of the patients (*n*=2/189). Revision surgery was performed in all these patients. Residual hump in 2 were observed and dorsum rasping was performed under local anesthesia. No tip revision was performed on any patient. Nasal tip movements were normal, and no stiffness was observed. Postoperative results of a patient are shown in Figs. 1a–m, 2a–m.

## Discussion

In our study, we aimed to reveal our techniques and clinical results on tip surgery in DPR. It is aimed to obtain a strong septum in the fixation made by preserving the perichondrium in the septum and leaving 2 mm of cartilage on the vomer and maxilla. An appropriate tip surgery is possible with a strong septum. The purpose of triangular septal extension grafts taken from the septum is not to lengthen the septum, but to prevent from the loss of tip projection and derotation seen in the late period. With the membranous tongue-in groove suture, a nasal tip that can move in all directions is aimed by avoiding hanging columella. With modified low septal strip septoplasty, which is mentioned in the surgical technique, a small amount of graft is taken from the septum, leaving a cartilage reserve for use if revision is required in the future. By loosening the ligaments in the columella with the help of scissors, anterior retraction in the philtral column is also prevented, after providing adequate type projection and rotation in patients with tension nose. There was no need for tip revision in the patients included in the study, and an effective tip surgery was performed with these combined techniques.

Septal extension grafts were used and recommended by Kosins and Daniel to correct underprojected tips [11]. In the study by Nakamura et al., an end to end septal extension graft is placed in the caudal septum in the open approach preservation rhinoplasty technique [12]. After the tip is reshaped, the preserved interdomal ligament is placed on the septal extension graft. This technique is called the interdomal hanger technique. They state that good aesthetic results were obtained. But the hanger technique is suitable for primary cases. We placed two triangular cartilage grafts symmetrically to the right and left sides of the septum, not extending far from the caudal septum and exceeding the dome height. Since we placed it symmetrically, asymmetry in the nasal tip was prevented. In addition, since overlapping was made, it was more stable than the direct extension technique and bending to the right and left was prevented [2, 5]. In the literature, a patient reported outcome measure questionnaire was conducted on the functional results of the septal extension graft, and it was stated that excellent aesthetic results were obtained without causing functional impairment [13].

The septal extension graft that we used in the study is not suitable for nasal lengthening. In case where nasal lengthening is main concern, nose surgeon needs to choose different techniques, such as spreader grafts, columellar strut grafts, other septal extension graft types and tongue in groove technique [14]. These techniques are used especially in Asian nose and secondary rhinoplasty [6, 15, 16]. In addition, ethmoid bone, alloplastic implant, costal and ear cartilage may be used for adequate lengthening. [3, 17, 18]

In our study, the septal extension graft was not visible from the outside, as it did not extend beyond the caudal septum and did not exceed the dome heights. Displacement of the septal extension graft may cause hanging columella [4]. For a robust septal extension graft, it is very important that the septum has adequate support [19]. We performed the least possible septal cartilage resection using the modified low septal strip septoplasty technique in our patients. This allowed us to obtain a stronger septum. Particularly since it is more effective in correcting the deviation of the septum, the low septal strip technique will provide strong septal support and reduce the problems seen in the nasal tip. Sen and Iscen described caudal septal advancement for tip support [20]. But this technique is not suitable for low septal strip septoplasty. Koçak and Gökler described auto-septal projection graft [9]. Excess cartilage is excised from the dorsum up to 1 cm to the caudal septum, leaving a cartilage like projection graft. But this technique is also not suitable for preservation rhinoplasty.

In the present study, the medial crus were stitched to the caudal septum and septal extension grafts using the tongue-in-groove technique. Concerns of the surgeons about the TIG technique are over the stiffness of the nasal tip. In the study conducted by Karaiskakis et al., 92.3% of the patients had nasal tip rigidity in the postoperative period [21]. We performed fixation by taking a very small bite from the cranial part of the medial crus. Thanks to the flexible septal extension grafts, we did not observe any nasal tip stiffness in our patients.

In the literature, columellar strut and septal extension graft were compared and there was a loss of projection and rotation in both in the late period, when the groups were compared, no statistically significant difference was found [22]. However, it was observed that loss of projection was less in patients with septal extension graft. In a study by Martinez et al., septal extension graft and columellar strut were compared and a significant decrease in tip rotation was observed in patients with columellar strut compared to patients with septal extension graft in the late period [23]. In a study by Sazgar et al., patients who underwent TIG alone and patients who underwent TIG with septal extension graft were compared and it was shown that tip rotation was preserved in the late period in both groups and that the septal extension graft did not affect the type rotation [24]. In our patients, we did not use columellar strut grafts. Because in our clinical experience and literature shows that, columellar strut grafts may cause hanging columella and dropping nose appearance in the late period.

The septal extension grafts we used are very similar to those used by Rohrich et al. and El Badawy and El Saloussy, but we harvested cartilage grafts from the base and caudal of the septal cartilage in accordance with the low septal strip technique [25, 26]. El Badawy and El Saloussy obtained good aesthetic results. The tip was not rigid, and the technique may be performed in the secondary rhinoplasty. The difference from our technique is that they leave L-strut behind. Our aim is to take a minimum cartilage graft from the septum and leave a large amount of cartilage if revision is required in the future. We observed that the L-strut can curve to the right and left. Therefore, we do not prefer leaving L-strut behind and we perform dorsal preservation rhinoplasty with modified low septal strip technique.

The limitations of the study are the absence of a control group and the short follow-up period. With the late results, the effectiveness of the techniques can be better evaluated. However, the present results show that an effective tip surgery has been achieved.

## Conclusion

A strong septal fixation is one of the most important factors affecting the success of tip surgery. A strong tip fixation is achieved with the modified low septal septoplasty technique described in this publication, and when combined with septal extension graft, tongue in groove technique and other suture techniques, an effective and permanent tip plasty can be performed in DPR.

## Supplementary Information

Below is the link to the electronic supplementary material.

Supplementary file1: 3d animation of the tip plasty. (MP4 3517 kb)

Supplementary file2: Surgical video is presented to show the surgical maneuvers in detail. (MP4 443276 kb)
